# Clinical Results of Knot-tying Versus Knotless Suture Anchors in Arthroscopic Anteroinferior Labral Repair

**DOI:** 10.7759/cureus.40292

**Published:** 2023-06-12

**Authors:** Andrea H Johnson, Jane C Brennan, Cyrus J Lashgari, Benjamin M Petre, Justin J Turcotte, Daniel E Redziniak

**Affiliations:** 1 Orthopedics, Anne Arundel Medical Center, Annapolis, USA; 2 Orthopedic Research, Anne Arundel Medical Center, Annapolis, USA; 3 Orthopedic Surgery, Anne Arundel Medical Center, Annapolis, USA

**Keywords:** arthroscopic bankart, shoulder arthroscopy, glenohumeral instability, knotless suture anchor, knot-tying suture anchor, anteroinferior labral tear

## Abstract

Background

Arthroscopic repair of glenohumeral instability is becoming an increasingly common procedure. These repairs can be undertaken using knot-tying and knotless suture anchors; there is currently no clear consensus in the literature about what type of repair is most cost-effective and provides superior outcomes. The purpose of this study is to examine postoperative outcomes of patients undergoing arthroscopic anteroinferior labral repair (AALR) with either knot-tying or knotless anchors.

Methods

A single institution retrospective observational cohort study of 122 patients undergoing AALR from January 2014 to June 2021 was conducted. Univariate statistics were used to assess differences in demographics, operative characteristics, and postoperative outcomes between repair types; multivariate analysis was used to evaluate risk factors for recurrent instability and reoperation.

Results

Patients undergoing AALR with knotless anchors had a shorter case duration than those with knot-tying anchors (112.64 vs. 89.86 minutes, p<0.001). There were no significant differences between groups in the size of labral tear, presence of a glenoid bone defect, or Hill-Sachs lesion. After controlling for age, BMI, sex, glenoid bone defect, number of preoperative dislocations, and fixation type, only age (OR=0.896, p=0.010) and female sex (OR=5.341, p=0.008) were independent risk factors for recurrent instability and no factors were independent predictors of reoperation.

Conclusion

Patients undergoing AALR experienced similar rates of reoperation and recurrent instability regardless of whether a knot-tying or knotless repair was performed. The use of knotless suture anchors may improve cost-effectiveness due to decreased surgical time without diminishing postoperative outcomes.

## Introduction

Glenohumeral instability (GHI) is a common condition that can cause significant pain and loss of function in both athlete and non-athlete populations [1]. The reported incidence of shoulder dislocations seen in United States emergency departments is 23.9 per 100,000 person-years, with younger individuals and males at higher risk of this type of injury [2]. Patients who experience one episode of instability are at significantly increased risk of recurrent instability without surgical intervention [1,3-5]. GHI encompasses a number of different pathologies including anterior-inferior or Bankart lesions, posterior-inferior or reverse Bankart lesions, superior labral (SLAP) injuries, pan-labral injuries, and multi-directional instability; each of these have unique considerations for surgical treatment [6]. Arthroscopic treatment of GHI is widely accepted and reliably leads to significant functional improvements for patients undergoing these procedures [7,8].

Early arthroscopic repairs for GHI involved the use of suture anchors using a knot-tying technique to secure the repair. Arthroscopic knot-tying is a technically difficult procedure that requires significant experience to master [9]. In 2001, the knotless suture anchor was introduced as an acceptable alternative to knot-tying suture anchors [10]. Since then technology has continued to progress with the addition of bio-absorbable suture anchors, all suture anchors and suture tape, each providing different benefits and drawbacks for GHI repair [11-13]. There is no consensus in the literature currently about which type of repair provides the best outcomes. Knotted anchors are often considered to produce a more secure, stronger repair, while knotless anchors are touted as having a smaller learning curve and improving postoperative shoulder function due to the lack of knots potentially interfering with motion [7,14]. Knot stacks may also be associated with postoperative cartilage damage secondary to abrasion [13]. The purpose of our study was to evaluate knot-tying and knotless suture anchor repairs for anteroinferior labral repairs by comparing rates of recurrent instability and reoperation along with postoperative functional outcomes between knot-tying and knotless repairs. To our knowledge, this is one of the largest studies to evaluate the differences between knot-tying and knotless suture anchors in anteroinferior GHI repair.

## Materials and methods

Study population and setting

This study was deemed institutional review board exempt by the institution’s clinical research committee. A retrospective observational study was conducted of patients undergoing arthroscopic anteroinferior instability repair from January 2014 to June 2021. Procedures were performed by eight board-certified attending orthopedic surgeons each with greater than 10 years of experience in a single hospital-based outpatient surgery center; no surgeries were performed by residents or fellows. Patients were included in the study if they had only an anteroinferior labral tear, and if the repair was performed with all knot-tying or all knotless anchors only. Patients were excluded from the study if they underwent open surgery or if they had a posteroinferior or SLAP repair or a capsular shift only, no other exclusion criteria were used. The average age of patients included was 25.01 years (range: 13-74 years), and the average time from injury to surgery was 9.04 months (range: 0.47-87.53 months). 

Procedures included and repair techniques

**Figure 1:**
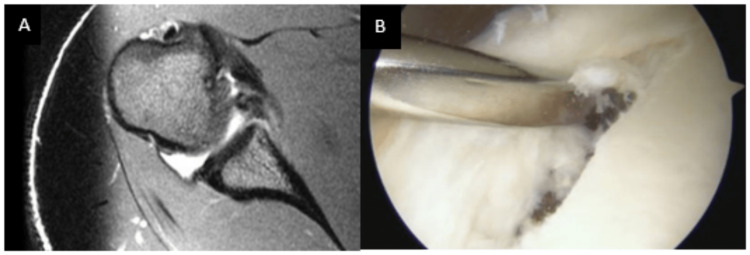
Preoperative and intraoperative images of anterior labral tears A. Axial T2 MRI of a right shoulder demonstrating a displaced anterior labral tear. B. Right shoulder displaced anterior labral tear in the lateral decubitus position when viewing from the anterior superior arthroscopic portal.

All arthroscopic anteroinferior (Bankart) repairs were included in this study. For all arthroscopic knot-tying types of repairs, suture anchors are placed sequentially in the glenoid at the site of the tear and then a suture shuttle device is used to shuttle the suture through the capsule/labral complex. Standard arthroscopic knot-tying techniques were utilized to obtain fixation. For knotless repairs, a suture shuttle device was used to shuttle the suture or tape through the capsule/labral complex. The suture or tape is then loaded into the knotless anchor and advanced using a mallet. Appropriate tension is obtained during anchor placement. Knot-tying repairs were all performed using sutures; knotless repairs were performed with sutures or suture tape. Preoperative magnetic resonance imaging (MRI) and an intraoperative arthroscopic view depicting a Bankart lesion are presented in Figure 1.

**Figure 2:**
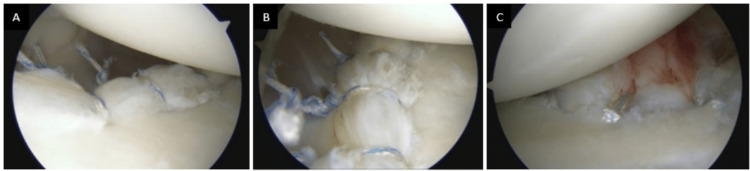
Intraoperative images of knotted and knotless repairs A. Right shoulder anterior labral repair with three knotted anchors in the lateral decubitus position when viewing from the posterior portal. B. Right shoulder anterior labral repair with three knotted anchors in the lateral decubitus position when viewing from the anterior superior portal. C. Right shoulder anterior labral repair with three knotless anchors in the lateral decubitus position when viewing from the posterior portal.

Intraoperative images depicting examples of knotted and knotless repairs are presented in Figure 2.

Perioperative protocol

All surgeries were performed on an outpatient basis under general anesthesia; regional anesthesia for postoperative pain control was used at the discretion of the surgeon and anesthesiologist. Surgeries were performed in either the seated position using a beach chair positioner or in the lateral position, based on surgeon preference and repair type. All patients were discharged with a sling/shoulder immobilizer, which is used for the first four weeks postoperatively. Patients received instruction for a home exercise program including hand, wrist, and elbow range of motion (ROM) and shoulder pendulums to begin the day after surgery, and formal physical therapy begins 14 days after surgery. Rehabilitation protocols advance from passive shoulder ROM starting at two weeks, to active assist ROM starting between four and six weeks and active ROM starting between six and eight weeks postoperatively; some restrictions in motion remain for up to 12 weeks postoperatively based on the repair type and quality. Return to sports occurs between four to six months postoperatively depending on the specific actions required; return to contact sports occurs no earlier than six months. 

Upper extremity functional score

The upper extremity functional score (UEFS) is a validated, reliable, eight-item tool that asks patients to evaluate their amount of difficulty performing tasks on the list on a scale of 1-10 [15]. It is used primarily by physical and occupational therapists to monitor progression through a rehabilitation program. At this institution, physical and occupational therapists administer this tool upon first evaluation of the patient and then repeat the measurement every four weeks until the patient is discharged. We recorded the first postoperative and last postoperative UEFS.

Data collection and analysis

Demographics, including age, BMI, and sex, and details of the injury and primary repair were manually recorded from the electronic medical record (EMR). Percent of labrum torn was calculated by the surgeon’s description of tear size based on the clock face description from the operative note. The number of preoperative dislocations was frank dislocations documented in the preoperative notes; subluxations or instability episodes without frank dislocation were classified as no dislocations. Glenoid bone defects and Hill-Sachs lesions were recorded if they were noted in the operative reports and/or on the preoperative MRI; the percentage of bone loss was not reliably available. The primary endpoints of the study were documented episodes of recurrent instability and reoperation on the ipsilateral shoulder. Univariate statistics (two-sided independent and paired samples t-tests, and chi-square tests) were used to assess differences in demographics, instability type, operative characteristics, and postoperative outcomes in patients by anchor type. Multivariate logistic regression was used to identify which factors independently contributed to recurrent instability and revision surgery. All statistical analysis was performed in SPSS version 27 (IBM, Armonk, NY) and statistical significance was assessed at α=0.05.

## Results

One hundred and twenty-two patients were included in this study. The average age of patients was 25.01±11.17 years (range 13-74 years), with a BMI of 26.83±6.03 kg/m^2^; 74.6% of patients were male. Only knot-tying suture anchors were used in 36 patients, while only knotless suture anchors were used in 86 patients. Thirty-nine patients completed some or all of their physical therapy at our institution and had UEFS recorded. The first postoperative UEFS was measured at an average of 2.82±2.02 weeks and the last postoperative UEFS was measured at an average of 16.36±7.79 weeks. The time to last known follow for all patients was 9.97±12.29 weeks.

Table 1 examines the differences in demographics, injury characteristics, and operative factors between patients undergoing repairs with knot-tying or knotless suture anchors.

**Table 1:** Preoperative and Operative Characteristic Differences Between Repairs with Knot-Tying vs. Knotless Anchors Data presented as n(%) or mean±SD; p≤0.05 in bold; ^a^30 patients, ^b^57 patients, ^c^22 patients, ^d^46 patients

	Knot-Tying Anchor N=36	Knotless Anchor N=86	P Value
Age, years	23.39±8.57	25.69±12.07	0.302
BMI, kg/m^2^	26.83±4.43	26.83±6.63	0.995
Male sex	31 (86.1)	60 (69.8)	0.059
Percent labrum torn, %	24.72 ± 6.37^a^	24.41 ± 5.42^b^	0.814
Number of dislocations preoperatively			
0	7 (19.4)	27 (31.4)	0.179
1	14 (38.9)	18 (20.9)	0.040
≥ 2	14 (38.9)	41 (47.7)	0.374
Glenoid bone defect	13 (36.1)	18 (20.9)	0.079
Hill-Sachs lesion	27 (75.0)	66 (76.7)	0.836
Time in operative room, minutes	112.64±25.41	89.86±21.09	<0.001
Number of anchors	3.00±0.76	2.98±0.61	0.859
Time from initial injury to surgery, months	10.78±19.31^c^	8.21±15.41^d^	0.557

There was a significant difference between anchor types in the number of minutes in the operating room (OR), with repairs using knot-tying anchors being significantly longer (112.64 vs. 89.86 minutes, p<0.001). Patients that underwent repairs with knotless anchors were less likely to experience only one preoperative dislocation (38.9 v. 20.9%, p=0.040). Table 2 examines the postoperative differences between patients undergoing repairs with knot-tying or knotless suture anchors; there were no significant differences between groups.

**Table 2:** Postoperative Characteristic Differences Between Repairs with Knot-Tying vs. Knotless Anchors Data presented as n(%) or mean±SD; *indicates the use of Fisher’s exact test; ^a^10 patients, ^b^29 patients, ^c^27 patients; UEFS, upper extremity functional score

	Knot-Tying Anchor N=36	Knotless Anchor N=86	P Value
First postoperative UEFS	27.30 ± 13.66^a^	38.52±20.57^b^	0.118
Last postoperative UEFS	68.30 ± 13.43^a^	72.52±8.10^c^	0.250
Change in UEFS	41.00 ± 11.00^a^	35.00±20.12^c^	0.380
Time to reinjury, months	23.88 ± 8.71	17.24±14.44	0.413
Time to revision, months	16.90 ± 10.53	24.24±13.06	0.403
Time to last follow-up, months	10.10 ± 11.69	9.92±12.60	0.942
Recurrent instability	4 (11.1)	15 (17.4)	0.379
Subsequent reoperation	3 (8.3)	9 (10.5)	1.000*

Both knot-tying (p<0.001) and knotless (p<0.001) anchor repair groups showed significant improvement in UEFS from the first postoperative evaluation to the last postoperative evaluation.

Table 3 evaluates which factors independently contribute to recurrent instability.

**Table 3:** Multivariate Regression Model for Recurrent Instability p≤0.05 in bold

	Odds Ratio	95% Confidence Interval	P Value
Age	0.896	0.824-0.974	0.010
Female sex	5.341	1.556-18.335	0.008
Glenoid bone defect	1.323	0.344-5.079	0.684
one preoperative dislocation	4.588	0.918-22.922	0.063
two or more preoperative dislocations	2.750	0.624-12.119	0.181
Knotless anchor	1.794	0.484-6.649	0.382

Age, sex, presence of a glenoid bone defect, number of preoperative dislocations, and repair type were included in the model. After controlling for all other factors, younger patients (OR=0.896, p=0.010) and female patients (OR=5.341, p=0.008) are more likely to experience recurrent instability. Table 4 evaluates which factors independently contribute to requiring subsequent reoperation.

**Table 4:** Multivariate Regression Model for Reoperation

	Odds Ratio	95% Confidence Interval	P Value
Age	0.955	0.887-1.027	0.211
Female, sex	2.115	0.539-8.304	0.283
Glenoid bone defect	1.351	0.314-5.813	0.686
one preoperative dislocation	1.814	0.351-9.369	0.477
two or more preoperative dislocations	1.162	0.255-5.301	0.846
Knotless anchor	1.378	0.326-5.837	0.663

After controlling all other factors, no factor was independently predictive of reoperation. Of the 12 patients that required reoperation during the study period, three patients underwent manipulation under anesthesia and arthroscopic lysis of adhesions for adhesive capsulitis, four underwent an arthroscopic revision of the instability repair, four underwent an open revision of instability repair, and one underwent an open coracoid process transfer.

## Discussion

There were significant differences in case duration and the number of patients experiencing only one preoperative dislocation between knot-tying and knotless anchors. All groups experienced statistically significant improvement in the UEFS from the first postoperative PT evaluation to the last postoperative PT evaluation, although there were no significant differences in the UEFS between groups at any time period. Consistent with other studies, the population was predominantly male [1,6].

Historically, arthroscopic GHI repairs with suture anchors were undertaken using a knot-tying construct to complete the repair [16,17]. This provided an overall successful result when compared with open surgery, and biomechanical studies have proven the structural integrity of this repair technique [6,18]. Despite the success of a knot-tying technique, there were some concerns as well. Knot-tying anchors leave a knot stack that can cause ongoing soft tissue irritation and cartilage abrasions, and studies have suggested that knot-tying anchors may damage microvascular circulation, increasing the risk of re-tear [13]. There are also studies showing a loss of motion with the use of knot-tying suture anchors in Bankart repairs [14,19]. Knotless suture anchors for arthroscopic GHI repair were introduced in 2001 as an alternative to knot-tying suture anchors [10]. Early on there was concern regarding the strength of the knotless repair construct. One early study by Cho et al. demonstrated an unsatisfactory result with regard to recurrent dislocation rate when comparing knot-tying to knotless suture anchors in arthroscopic Bankart repair with 4.9% of the knot-tying group and 23.8% of the knotless group experiencing recurrent dislocation [7]. A contemporary study by Garofalo et al. showed a more favorable 5% recurrent dislocation rate for knotless repairs [12]. When looking at anteroinferior repairs in this study, our overall recurrent instability rate was higher at 15.6%, although there was no significant difference when comparing repair types. More recent studies have shown little or no difference in repair strength or recurrent dislocation between anchor types in both clinical and biomechanical studies [8,14,18]. Patients in the current study who underwent repairs with knotless anchors had a higher rate of recurrent instability and reoperation though this was not statistically significant; fixation type was not an independent predictor of recurrent instability or reoperation. Similar to the more recently published studies, we did not see any significant differences in recurrent instability episodes or reoperation between patients who had repairs with knot-tying anchors or knotless anchors. 

A recent study by Wu et al. found a small but significant improvement in forward elevation in patients undergoing anteroinferior repair with knotless suture anchors, but no differences in internal or external rotation [14]. In the current study, knotless anchors were used with greater frequency than knot-tying anchors. While we did not look specifically at postoperative ROM, the potential ROM restriction may contribute to the preference for knotless repairs. While maximizing functional outcomes is important, and the selection of anchor type may contribute to patients’ functional improvement, numerous studies have shown consistent improvements in functional outcomes for all patients undergoing arthroscopic treatment of shoulder instability regardless of the type of anchor used [9,20-22]. The current study measured postoperative function using the UEFS, which is a tool that looks at global function rather than sports-specific function. We demonstrated significant improvement from the initial postoperative assessment to the final postoperative assessment among all patients and no significant difference between the types of anchors used.

Arthroscopic knot tying is a skill with a significant learning curve and can be challenging for even the most experienced arthroscopic surgeon [23,24]. Knotless suture anchors provide a more consistent result with a shorter learning curve [10,13]. Due to the increased difficulty of tying arthroscopic knots, it has been hypothesized that GHI repairs with knotless anchors may result in a shorter case duration. In a study by Kocaoglu et al., a significant difference in case duration between the two types of anchors was observed, with repairs using knotless anchors being significantly shorter than repairs with knot-tying anchors [20]. In the current study patients undergoing repairs with knotless anchors had a case duration that was more than 22 minutes shorter than those with knot-tying anchors. While this decrease in case duration may have minimal clinical impact, OR time has been cited to cost approximately $24/minute and saving even minutes of surgical time can help control costs and improve our ability to provide high-quality, lower-cost healthcare [25,26].

This study does have a number of limitations. First, it is a retrospective study and there is an inherent selection bias. Second, the study was performed at a single institution, therefore its findings may not be generalizable to the larger patient population. However, all participating surgeons are highly experienced arthroscopic surgeons and utilize similar pre- and postoperative protocols that may reduce confounding from variability in care processes. Third, due to the age group that typically undergoes this type of surgery and the potentially transient nature of this population, there may have been additional episodes of recurrent instability or reoperation that were performed at outside institutions and not accounted for in our data; this also limited our availability of UEFS data. We also were unable to account for additional patient factors such as sport-specific factors that could have contributed to a higher risk of recurrent instability and need for revision surgery, although we did evaluate multiple injury factors and found no significant differences between groups. Further studies should investigate patient-reported outcomes and quality of life of patients undergoing these procedures.

## Conclusions

Both knot-tying and knotless suture anchor repairs for GHI provide similar results for patients undergoing these arthroscopic procedures; neither injury type nor fixation type was independently predictive of recurrent instability or reoperation. The use of knotless suture anchors resulted in shorter case duration for anteroinferior repairs and thus may improve the cost-effectiveness of the surgical procedure without diminishing postoperative patient outcomes.
